# Object Detection in Laparoscopic Surgery: A Comparative Study of Deep Learning Models on a Custom Endometriosis Dataset

**DOI:** 10.3390/diagnostics15101254

**Published:** 2025-05-15

**Authors:** Andrey Bondarenko, Vilen Jumutc, Antoine Netter, Fanny Duchateau, Henrique Mendonca Abrão, Saman Noorzadeh, Giuseppe Giacomello, Filippo Ferrari, Nicolas Bourdel, Ulrik Bak Kirk, Dmitrijs Bļizņuks

**Affiliations:** 1 Institute of Applied Computer Systems, Riga Technical University, LV-1048 Riga, Latvia; andrejs.bondarenko@rtu.lv (A.B.); vilens.jumutcs@rtu.lv (V.J.); 2 Department of Obstetrics and Gynecology, Marseille Hospital, 13005 Marseille, France; antoine.netter@ap-hm.fr (A.N.); fannyduch@outlook.fr (F.D.); 3 Gynecologic Division, Beneficência Portuguesa de São Paulo, Sao Paulo 01323-001, Brazil; hmabrao@gmail.com; 4 SurgAR, 63000 Clermont-Ferrand, France; saman.noorzadeh@surgar-surgery.com (S.N.); giuseppe.giacomello@poliambulanza.it (G.G.); filippoalberto.ferrari@sacrocuore.it (F.F.); nicolas@surgar-surgery.com (N.B.); 5 Department of Obstetrics and Gynecology, Istituto Ospedaliero Fondazione Poliambulanza, 25124 Brescia, Italy; 6 Department of Obstetrics and Gynecology, Gynecologic Oncology and Minimally Invasive Pelvic Surgery, International School of Surgical Anatomy (ISSA), IRCCS “Sacro Cuore—Don Calabria” Hospital, Negrar di Valpolicella, 37024 Verona, Italy; 7 Department of Clinical Research and Innovation, CHU Clermont Ferrand, 63100 Clermont-Ferrand, France; 8 Department of Public Health, Aarhus University, 8000 Aarhus, Denmark; ubk@ph.au.dk; 9 The Research Unit for General Practice, 8000 Aarhus, Denmark

**Keywords:** endometriosis, deep learning, object detection, RCNN

## Abstract

**Background:** Laparoscopic surgery for endometriosis presents unique challenges due to the complexity of and variability in lesion appearances within the abdominal cavity. This study investigates the application of deep learning models for object detection in laparoscopic videos, aiming to assist surgeons in accurately identifying and localizing endometriosis lesions and related anatomical structures. A custom dataset was curated, comprising of 199 video sequences and 205,725 frames. Of these, 17,560 frames were meticulously annotated by medical professionals. The dataset includes object detection annotations for 10 object classes relevant to endometriosis, alongside segmentation masks for some classes. **Methods:** To address the object detection task, we evaluated the performance of two deep learning models—FasterRCNN and YOLOv9—under both stratified and non-stratified training scenarios. **Results:** The experimental results demonstrated that stratified training significantly reduced the risk of data leakage and improved model generalization. The best-performing FasterRCNN object detection model achieved a high average test precision of 0.9811 ± 0.0084, recall of 0.7083 ± 0.0807, and mAP50 (mean average precision at 50% overlap) of 0.8185 ± 0.0562 across all presented classes. Despite these successes, the study also highlights the challenges posed by the weak annotations and class imbalances in the dataset, which impacted overall model performances. **Conclusions:** In conclusion, this study provides valuable insights into the application of deep learning for enhancing laparoscopic surgical precision in endometriosis treatment. The findings underscore the importance of robust dataset curation and advanced training strategies in developing reliable AI-assisted tools for surgical interventions. The latter could potentially improve the guidance of surgical interventions and prevent blind spots occurring in difficult to reach abdominal regions. Future work will focus on refining the dataset and exploring more sophisticated model architectures to further improve detection accuracy.

## 1. Introduction

Endometriosis is a chronic condition affecting an estimated 5–10% of women and adolescents of reproductive age (15–49 years). Recent data indicate an incidence rate of 2.37–2.49 per 1000 women per year, translating to an approximate prevalence of 6–8% [1]. The condition frequently goes undiagnosed or misdiagnosed, with an average delay of 4–11 years between the onset of symptoms and definitive diagnosis. While endometriosis can occur at any age, it predominantly affects women between menarche and menopause, peaking between the ages of 25 and 45 years. Notably, the prevalence is higher among individuals experiencing infertility and chronic pelvic pain [2].

This disease is characterized by the presence of endometrial-like tissue outside the uterus, leading to debilitating symptoms and complications, including infertility. The complexity of endometriosis and the variability in lesion appearances often pose significant challenges for diagnosis and treatment. Laparoscopic surgery, a minimally invasive technique, is considered the gold standard for both the diagnosis and management of endometriosis. Despite its advantages, such as reduced recovery times and minimal scarring, the procedure is hindered by the intricate and variable nature of the disease, necessitating high levels of expertise to accurately identify and treat lesions.

Recent advances in artificial intelligence (AI) have shown promise in addressing some of these challenges. Review studies [3,4,5] have demonstrated the effectiveness of machine learning and deep learning in enhancing diagnostic accuracy, optimizing clinical workflows, and supporting decision making in areas like drug discovery, oncology, radiology, and genomics. These applications are not only highlighting the transformative potential of AI in healthcare but also broadening the understanding of its diverse and evolving impact.

In particular, deep learning models in object detection, offer the potential to assist surgeons in identifying and localizing endometriosis lesions and related anatomical structures during laparoscopic procedures. A custom dataset curated for this purpose, comprising of 199 laparoscopic videos with over 205,725 frames and extensive annotations by medical professionals, was utilized to explore the application of various AI techniques. These approaches include well-known object detection algorithms such as FasterRCNN [6] and YOLOv9 [7], along with custom encoder–decoder segmentation networks employed not to yield segmentation masks as the final output, but rather, to facilitate bounding box creation from the segmentation results.

This study emphasizes the importance of addressing challenges such as class imbalances through tailored augmentation techniques and loss functions. By leveraging these innovations, the research aims to establish a robust framework capable of adapting to the complexities of laparoscopic surgery. The integration of AI into surgical practice has the potential to enhance diagnostic precision, improve surgical outcomes, and, ultimately, contribute to better patient care.

## 2. Related Work

### 2.1. Existing AI Applications

Up until recent times, research mostly focused on diagnosing endometriosis via ultrasound imaging or MRI imaging [8], or via biomarker or metabolite analysis (see review in [9]). Recent research tackles the problem of endometrioma image classification [10] and detection [11]. These image analysis-based applications focus on using deep learning networks with convolutional backbones for object detection or segmentation tasks.

### 2.2. Review of Existing Datasets

Several datasets have been developed to support research in laparoscopic surgery image analysis, though most are not specifically tailored to the challenges posed by endometriosis.

Among the available data resources in this domain, some provide extensive pixel-level annotations of numerous anatomical structures extracted from surgical videos. These annotations ensure a rich representation of organs and tissues across thousands of frames [12]. Others concentrate on a wide range of abdominal organs and vessels, offering large, high-quality annotations that are valuable for general surgical data science but lack targeted pathological focus [13]. There are resources specifically designed for particular conditions, such as endometriosis, which include annotated images that are directly relevant to this pathology [14]. However, the number of such annotated images often remains limited, and the specificity of covered lesions can restrict broader applicability. Another innovative approach combines real surgical footage with synthetic data generated through semantic image synthesis, improving segmentation performance and enabling extensive experimentation with cutting-edge models [15].

Despite these advancements, challenges remain. Some datasets focus narrowly on certain procedures or do not capture the unique complexities of endometriosis, while others rely heavily on synthetic data that may not fully translate into clinical practice. Each resource, therefore, presents a mix of outstanding features—such as comprehensive organ annotations or innovative data augmentation methods—and notable issues, including insufficient coverage of specific conditions, limited annotated samples, or potential gaps in real-world applicability. These datasets, while valuable, do not fully address the unique challenges posed by endometriosis in laparoscopic surgery, particularly the need for comprehensive annotations across a wide variety of lesion types and surgical scenarios.

### 2.3. Limitations of Existing Datasets

The primary limitations of the existing datasets include small sample sizes, limited annotations, and a focus on either general anatomical structures or specific types of surgical procedures. For instance, while CholecSeg8K [12] and the Dresden Surgical Anatomy Dataset [13] offer detailed segmentations, they do not cover the pathological conditions relevant to endometriosis. On the other hand, GLENDA [14], while directly addressing endometriosis, is limited by the number of annotated frames and the variability in lesion types represented.

These limitations underscore the need for a new dataset that provides detailed, endometriosis-specific annotations across a broad range of surgical scenarios. Such a dataset would better support the development of AI models capable of assisting in the diagnosis and treatment of this complex condition.

## 3. Materials and Methods

This section introduces our custom endometriosis dataset and corresponding deep learning methodology to tackle the aforementioned object detection problem in laparoscopic images. First, the dataset collection, composition, and annotation details are described. Then, the machine and deep learning methodology along with the experimentation setup are outlined.

### 3.1. Endometriosis Dataset

#### 3.1.1. Overview

To address the complex challenge of object detection in laparoscopic videos for the treatment of endometriosis, we curated a comprehensive dataset specifically designed to support the development and evaluation of deep learning models. This dataset is meticulously tailored to facilitate the detection and classification of various endometriosis lesions and associated anatomical structures, providing a robust foundation for advancing AI-driven surgical tools.

#### 3.1.2. Data Collection and Composition

The dataset is comprised of carefully chosen 199 laparoscopic videos, resulting in a total of 205,725 frames. Of these, 17,560 frames have been meticulously annotated by experienced medical professionals. For the object detection task, annotations were focused on short sequences, typically consisting of 10–11 consecutive frames, while longer frame sequences were left unannotated, to ensure detailed and accurate labelling where it is most needed. This selective approach was adopted to effectively manage the large volume of data while capturing critical surgical moments and ensuring high-quality annotations.

The videos in the dataset were recorded at varying resolutions, reflecting the diverse conditions encountered during laparoscopic procedures. The majority of the videos were captured at a resolution of 1920 × 1080 pixels, providing high-definition imagery crucial for accurate annotation and model training. The distribution of video resolutions is summarized in Table 1.

#### 3.1.3. Annotation Details

Annotations within the dataset for object detection tasks are provided in the form of bounding boxes and segmentation regions, covering 10 distinct classes relevant to endometriosis and other anatomical structures. These classes include various types of adhesions, endometriomas, and superficial lesions, which are critical for surgical decision making. The distribution of annotated objects across these classes is detailed in Table 2.

The varying frequency of these object classes highlights potential challenges in model performance, particularly concerning class imbalance. It is crucial to address this imbalance to ensure that the models can effectively detect both common and rare conditions, thus improving their robustness and generalizability in real-world scenarios.

#### 3.1.4. Video Characteristics

**Figure 1:**
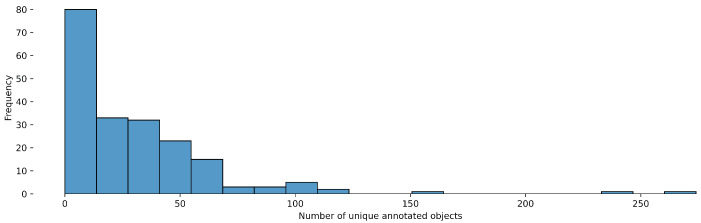
Object detection distribution of the number of unique annotated objects per video.

**Figure 2:**
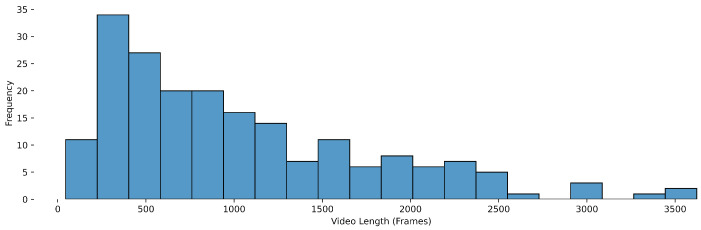
Object detection distribution of video lengths (in number of the frames).

The dataset includes videos of varying lengths, which contributes to the diversity and complexity of the data. This variation mirrors real-world surgical scenarios, where the duration of procedures can significantly differ depending on the complexity of the case and the surgeon’s expertise. The distribution of the number of unique annotated objects per video can be found in Figure 1. The distribution of video lengths, measured in the number of frames, provides an insight into the range of surgical cases represented within the dataset. The latter can be found in Figure 2.

Frames were carefully extracted at key surgical moments to ensure that the annotated frames capture critical events and relevant anatomical structures. The high resolution and clarity of these frames provide a solid foundation for training and evaluating object detection models, facilitating the development of tools that can assist surgeons in making precise and timely decisions during surgery.

#### 3.1.5. Data Organization and Accessibility

The dataset is organized into a well-structured directory system that enhances accessibility and usability. Each video is stored in a dedicated folder, containing both the raw video frames and the corresponding annotations. The annotations are stored separately with a clear naming convention that indicates the class and type of annotation (bounding box), allowing researchers to quickly locate and utilize the relevant data for their specific tasks. This organizational structure is designed to streamline the research process and ensure that the data can be efficiently integrated into various machine learning workflows.

#### 3.1.6. Considerations and Challenges

While this dataset provides a valuable resource for the development of object detection models in laparoscopic surgery, it poses certain challenges. One of the primary challenges is the variability in interpretation during the annotation process, despite being conducted by experienced medical professionals. This can lead to potential inconsistencies in the labels, which may affect model performance. Moreover, the proprietary nature of the dataset limits its broader distribution, which may restrict its use in the wider research community. This limitation could pose challenges for collaborative research efforts and the reproducibility of findings.

Another significant challenge is the class imbalance within the dataset. Certain lesion types are underrepresented, which could bias the models toward detecting more common conditions at the expense of rarer ones. Addressing this imbalance through techniques such as data augmentation, class re-weighting, or synthetic data generation will be crucial for developing models that are both robust and generalizable across diverse surgical scenarios.

### 3.2. Methods

In this section, we describe the methodology employed for training and evaluating object detection models on our laparoscopic endometriosis dataset. The primary focus is on comparing the performance of the FasterRCNN [6] and YOLOv9 [7] object detection models using both stratified and non-stratified data splits [16]. We also discuss the unique challenges posed by the dataset, such as annotation inconsistencies and class imbalance, and how we addressed them.

### 3.3. Data Preprocessing

The laparoscopic endometriosis dataset consists of high-resolution videos, with the frames mostly captured at 1920 × 1080 resolution. To preserve as much data as possible, we opted for the usage of this resolution for the FasterRCNN model. However, for the YOLOv9 model, we downscaled images to 640 × 640 pixels as it is a default model resolution which would produce a near real-time inference.

Bounding boxes were resized proportionally to match the new image dimensions, and 5 stratified data splits [16] were generated to ensure that all classes were represented equally in the train, validation, and test sets. Stratification was achieved using Python’s iterative-stratification package of version 0.1.7 accessed on 1 December 2024 (https://github.com/trent-b/iterative-stratification), which supports various data splitting strategies for multi-label scenarios. To prevent any data leakage, we split videos given multiple labels per example into train/test/validation sets; thus, entire video frames from the train set videos never appear in the validation and test sets. A failure to prevent common data leakages can pave the way to unsatisfactory results (undetected model overfitting) [17]. Additionally, we opted for exploring the effects of a data leakage by creating non-stratified data splits. We achieve the latter by introducing a data leak in between the train and validation datasets, but not in the train/test or validation/test sets.

### 3.4. Object Detection Models

We employed two object detection models—FasterRCNN and YOLOv9—to identify and classify endometriosis lesions and other relevant anatomical structures.

1. **FasterRCNN**: We used a FasterRCNN model with a ResNet50 backbone, initialised with pretrained weights from the ImageNet1K_V2 model in PyTorch’s (version 2.3.0) torchvision (version 0.18.0) model zoo. The model has 41.34 M parameters out of which 25.6 M parameters are attributed to the ResNet50 backbone. This model is a two-stage object detection architecture. It combines the aforementioned ResNet50 backbone with the Feature Pyramid Network (FPN) [18] which adds up lateral connections and top-down pathways as well as enhances a multi-scale feature representation. The final stage is represented by a Region Proposal Network (RPN) [19] which takes the feature map and proposes candidate object bounding boxes. FasterRCNN was trained with heavy augmentations to handle a high variability in the dataset.
2. **YOLOv9**: We utilized the largest of the V9 family: the YOLOv9e (https://docs.ultralytics.com/models/yolov9/#performance-on-ms-coco-dataset) model (accessed on 1 December 2024) with 58.1 M parameters, offering the highest accuracy and known for its extensive architecture as well as high performance on object detection tasks. The YOLOv9 model is composed of a backbone: the Generalized Efficient Layer Aggregation Network (GELAN); a neck: Programmable Gradient Information (PGI); and a head: the DualDDetect module. The model was pretrained on the COCO dataset [20] and supports 640 × 640 resolution inputs. It was fine-tuned on our dataset with the default Ultralytics augmentations: https://docs.ultralytics.com (accessed on 1 December 2024).

Each model was evaluated on the stratified and non-stratified data splits to compare their performances in the scenarios with varying degrees of a data leakage. The model, training, and evaluation code are available here: https://github.com/abvp200/female (accessed on 1 May 2025).

### 3.5. Training Strategy

#### 3.5.1. Stratified vs. Non-Stratified Splits

To ensure robust results, we performed experiments with both stratified and non-stratified splits. In stratified splits, frames from the same video were placed in the same subset (train, validation, or test) to prevent any data leakage. In non-stratified splits, train and validation frames were randomly sampled from the same videos, while test frames were held out from separate videos. This allowed us to compare performance in a more realistic setup and evaluate the risk of overfitting.

#### 3.5.2. Augmentation Techniques

Given the limited variability in the dataset (199 videos), heavy augmentations were crucial to improving model performance and preventing overfitting. For the FasterRCNN model, augmentations included Resize, Normalise, CenterCrop, and RandomFlip. Additionally, we implemented a custom augmentation pipeline, which resizes bounding boxes accordingly to maintain label consistency. The most important augmentation used for FasterRCNN only was the so-called Copy-Paste augmentation [21] used by the authors of the YoloV9 model. For the YOLOv9 model, we used default Ultralytics augmentations, including rotations, flips, mosaic, and colour augmentations. Generally speaking, the most advanced augmentations are considered to be the Copy-Paste one (used in our experiments with FasterRCNN) and the mosaic augmentation, which extends the MixUp augmentation [22].

### 3.6. Experimental Setup

In our experiments, we used several models with consistent training setups. The FasterRCNN model was trained with a batch size of 8 and the Adamax optimizer [23] (learning rate of 0.0001, betas set to (0.9, 0.999), epsilon 1 × $10^{-8}$, and no weight decay) over 125 epochs. Finally, the YOLOv9 model was trained with a batch size of 16, using the Adam optimizer [24] with default values for 5 epochs.

#### Evaluation Metrics

To evaluate the performance of our object detection models, we utilized several key metrics:

1. **Precision**: measures the proportion of correctly predicted positive instances among all predicted positives.
2. **Recall**: reflects the model’s ability to identify true positives (actual lesions).
3. **mAP50**: evaluates localization accuracy at an Intersection over Union (IoU) threshold of 0.50.
4. **mAP50-95**: assesses mean Average Precision over a range of IoU thresholds (0.50 to 0.95).
5. **Fitness**: a combined metric that balances precision and recall, often represented by the F-1 score.

For all metrics, we report the mean values alongside standard deviations to provide insights into the consistency and stability of the models’ performance across different data splits (stratified and non-stratified). The precision, recall, and mAP scores allow us to assess the strengths and weaknesses of each model in detecting and segmenting endometriosis lesions, while the fitness metric gives an overall perspective on the models’ reliability.

## 4. Main Results

**Figure 3:**
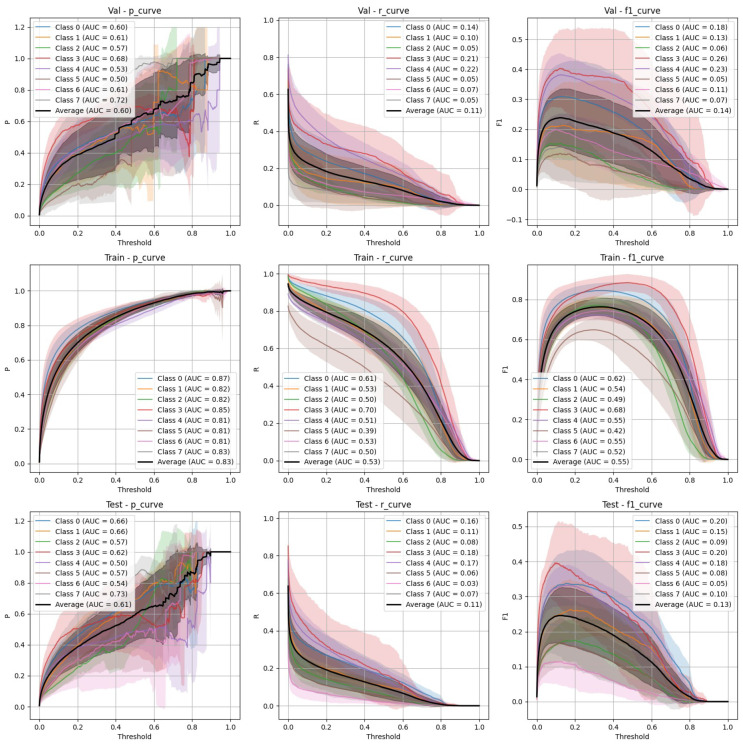
YOLOv9 stratified train/validation/test precision, recall, and F-1 curves.

**Figure 4:**
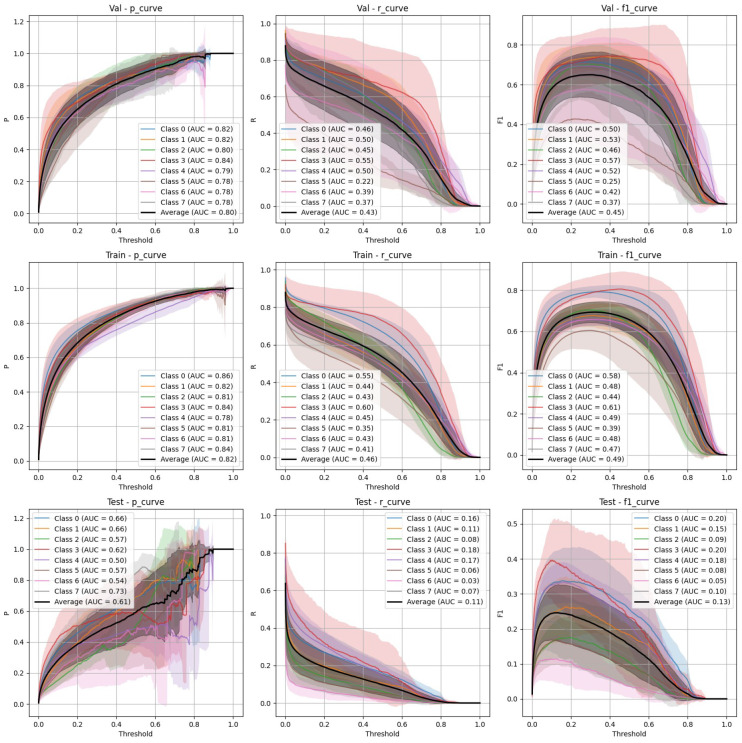
YOLOv9 non-stratified train/validation/test precision, recall, and F-1 curves.

**Figure 5:**
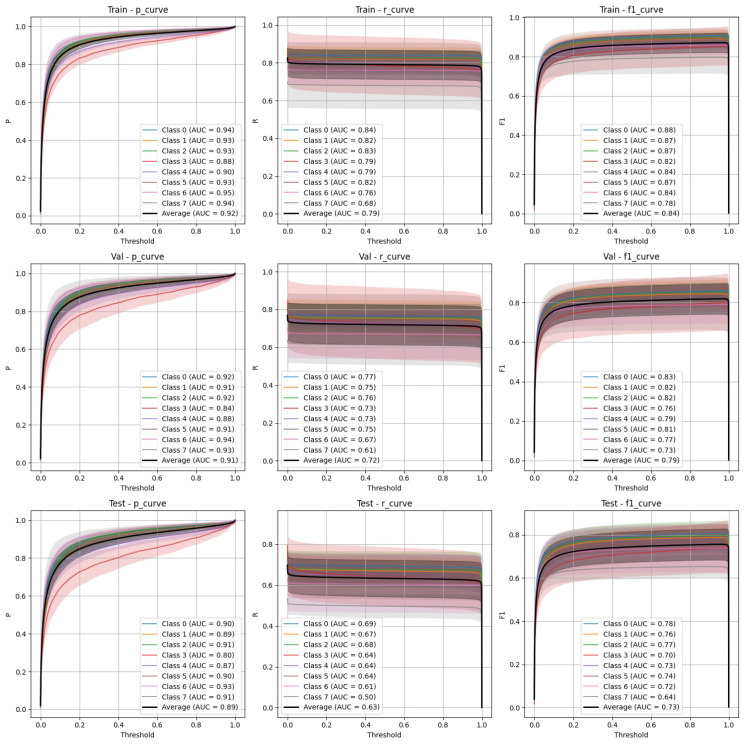
FasterRCNN stratified train/validation/test precision, recall, and F-1 curves.

**Figure 6:**
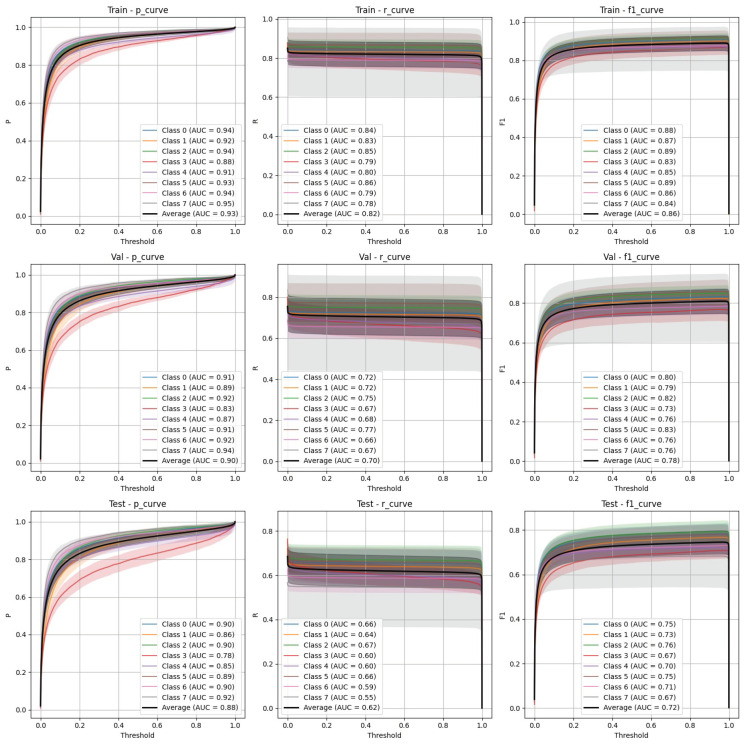
FasterRCNN non-stratified train/validation/test precision, recall, and F-1 curves.

**Figure 7:**
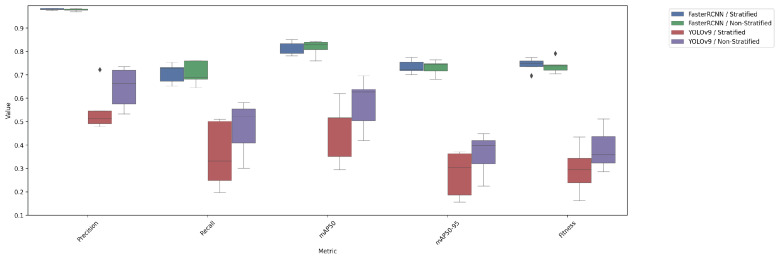
Test performance metrics by model and split.

The performance of YOLOv9 and FasterRCNN is analysed across both stratified and non-stratified splits, with particular attention to precision, recall, and mIoU scores. In general, FasterRCNN outperformed YOLOv9 in terms of overall precision and recall, with YOLOv9 showing more variability, especially in the stratified splits. Precision–recall and F-1 score curves highlight these trends, with FasterRCNN maintaining high precision and recall across the train, validation, and test sets. The main findings are outlined in Figure 3, Figure 4, Figure 5, Figure 6 and Figure 7.

### 4.1. YOLOv9 Performance

1. **Stratified split** (Figure 3): YOLOv9 showed lower precision and recall compared to FasterRCNN, with substantial variation in the stratified scenario. The F-1 curves demonstrate challenges in maintaining a balance between precision and recall, particularly on the validation and test sets.
2. **Non-stratified split** (Figure 4): YOLOv9 improved its performance in the non-stratified case, as reflected in its higher precision, recall, and mAP scores. The non-stratified F-1 curves also show better consistency across the training and validation sets.

### 4.2. FasterRCNN Performance

1. **Stratified split** (Figure 5): FasterRCNN demonstrated superior performance across all metrics in the stratified scenario, with precision exceeding 0.97 and relatively stable recall. The mAP50 and mAP50-95 scores indicate that the model was able to detect and segment objects with high accuracy.
2. **Non-stratified split** (Figure 6): While the performance of FasterRCNN remained high in the non-stratified scenario, slight differences were observed in precision and recall across the training, validation, and test datasets. F-1 curves for FasterRCNN consistently showed better generalization and balance across all splits compared to YOLOv9.

### 4.3. Training Challenges

**Figure 8:**
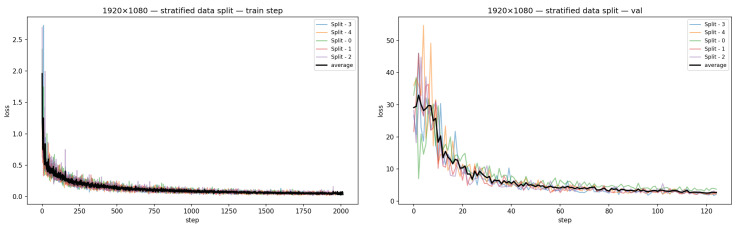
FasterRCNN train (**left**) and validation (**right**) loss convergences at the stratified split.

Several challenges were encountered during training. Overfitting was the main problem for all models. YOLOv9 showed signs of overfitting after just five epochs, likely due to the small dataset size in relation to the complexity of the model. Hence, we also decided against using Transformer-based models (e.g., ViT [25]) due to their propensity for overfitting on small datasets. To underpin our findings, we present two loss convergence graphs. Figure 8 represents training vs. validation loss convergences at the stratified split scenario for the FasterRCNN model on different folds as well as on average. As we can see from the presented figure, the final loss values on the validation set folds are still far away from the corresponding training loss values. In particular, we observe the final average training loss of 0.0589±0.0512 and the corresponding validation loss of 2.6983±0.5756 for the FasterRCNN model.

### 4.4. Comparison of Test Performance Metrics

Table 3 summarizes the test performance metrics for both YOLOv9 and FasterRCNN, highlighting the key differences between stratified and non-stratified training splits. FasterRCNN consistently outperforms YOLOv9 in both setups, particularly in terms of precision and mAP scores. The FasterRCNN model delivers the best average mAP score of 0.8185±0.0562 at stratified data splits compared to only a 0.5771±0.2113 average mAP score for the YOLOv9 model at non-stratified splits. To underpin our key findings in Table 3, we show an additional box plot representing the actual distribution of our metrics across five generated data splits in Figure 7.

### 4.5. Visual Validation and Results

**Figure 9:**
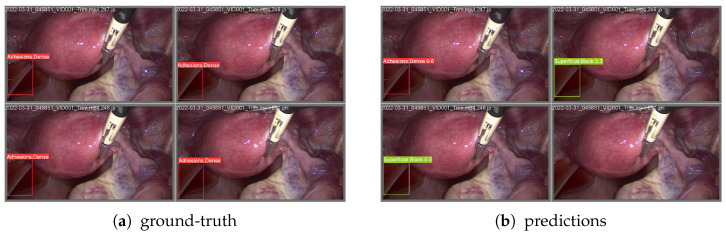
YOLOv9 ground-truth and predicted bounding boxes with the corresponding classes.

**Figure 10:**
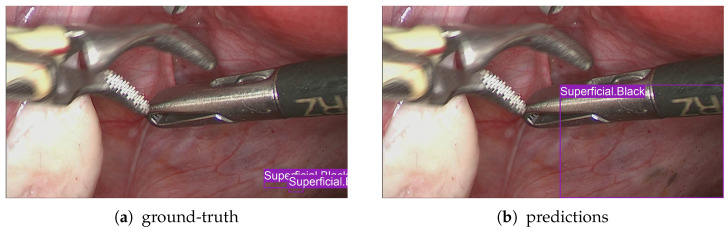
FasterRCNN ground-truth and predicted bounding boxes with the corresponding classes.

In this section, we present visual evidence of the performance achieved by both models—FasterRCNN and YOLOv9—on the typical frames from our proprietary dataset. As we can see from Figure 9 and Figure 10, FasterRCNN produces more aligned (with the ground-truth) matches of the annotated bounding boxes. In general, we observe fewer complete misses and non-predicted ground-truth classes for the FasterRCNN model than for the YOLOv9 model.

## 5. Discussion of Challenges and Future Directions

Both YOLOv9 and FasterRCNN faced challenges in handling class imbalances and potential overfitting, particularly in the stratified splits. YOLOv9’s performance was more sensitive to these factors, especially in terms of the recall, where it missed a significant number of true positives. On the other hand, FasterRCNN, while more consistent, also faced challenges in detecting smaller or more complex objects. Overfitting was an issue for YOLOv9, with validation loss increasing after just a few epochs. It is worth noting that class 8, “Superficial White”, was rather poorly represented in the training set and the model was not able to make any predictions out of it—thus, it is not present in Figure 3, Figure 4, Figure 5, Figure 6 and Figure 7. We observed more severe data leakage in the YOLOv9 model than in FasterRCNN, probably due to the smaller YOLOv9 model size and lower input image resolution. It failed to build robust visual features and their hierarchy and, therefore, was overfitted more severely and underperformed in comparison to the FasterRCNN-ResNet50 fine-tuned model.

We argue that potential real-time surgical application of the YOLOv9 model does not outweigh an observed poorer generalization performance in terms of reported metrics. Hence, one cannot tolerate missing problematic abdominal blind spots at lower input image resolutions. Finally, we argue that the FasterRCNN model can approach the inference speed of the YOLOv9 model (i.e., 30 FPS) at much higher input image resolutions [26] and the latest GPU cards.

To address all the aforementioned challenges, future work should explore data augmentation techniques as well, such as oversampling or synthetic data generation for underrepresented classes. Moreover, advanced model architectures, such as multi-target architectures using a mixture of datasets and tasks, could improve performance as well as pave the way for the Transformer-based approaches (due to an increase in the acquired training set size), which could further enhance generalization and performance.

## 6. Conclusions

This study provides an evaluation of deep learning models for object detection in laparoscopic surgery, focusing on endometriosis-specific challenges. Among the tested models, FasterRCNN demonstrated the best performance in both stratified and non-stratified setups, showcasing its potential as a robust tool for detecting complex anatomical structures and lesions in laparoscopic videos. YOLOv9, while competitive, exhibited variability and a higher susceptibility to overfitting, emphasizing the importance of data quality and model architecture. The challenges identified, including class imbalance and data leakage, underline the critical need for rigorous dataset preparation and advanced augmentation techniques. Future work will focus on overcoming these limitations by incorporating additional data, exploring advanced model architectures, and refining augmentation strategies. Ultimately, these efforts aim to bridge the gap between experimental results and real-world surgical applications, paving the way for AI-driven improvements in endometriosis diagnosis and treatment.

## Figures and Tables

**Table 1:** Video resolutions.

Resolution	Number of Videos
1920 × 1080	193
1280 × 720	5
720 × 576	1

**Table 2:** Class distribution for object detection.

Class Name	Class ID	Number of Annotated Objects
Adhesions Dense	0	1424
Adhesions Filmy	1	537
Deep Endometriosis	2	700
Ovarian Chocolate Fluid	3	223
Ovarian Endometrioma	4	302
Ovarian Endometrioma[B]	4	382
Superficial Black	5	835
Superficial Red	6	642
Superficial Subtle	7	509
Superficial White	8	463

**Table 3:** Test performance metrics with standard deviations for YOLOv9 and FasterRCNN (stratified and non-stratified data splits).

Model/Split	Precision	Recall	mAP50	mAP50-95	Fitness
FasterRCNN/stratified	0.9811 ± 0.0084	0.7083 ± 0.0807	0.8185 ± 0.0562	0.7345 ± 0.0554	0.7429 ± 0.0555
FasterRCNN/non-stratified	0.9787 ± 0.0107	0.7076 ± 0.0957	0.8162 ± 0.0647	0.7309 ± 0.0612	0.7395 ± 0.0615
YOLOv9/stratified	0.5504 ± 0.1864	0.3580 ± 0.2701	0.4599 ± 0.2503	0.2767 ± 0.1877	0.2951 ± 0.1939
YOLOv9/non-stratified	0.6458 ± 0.1662	0.4742 ± 0.2193	0.5771 ± 0.2113	0.3622 ± 0.1656	0.3837 ± 0.1701

## Data Availability

Restrictions apply to the availability of these data. Data was obtained from SurgAR and are available from the authors with the permission of SurgAR.
